# The role of astragalus polysaccharides in alleviating transport stress in piglets

**DOI:** 10.1371/journal.pone.0356186

**Published:** 2026-09-08

**Authors:** Peng Liu, Jinjiao Zuo, Hui Lu, Caihong Wu

**Affiliations:** Jiangsu Agri-Animal Husbandry Vocational College, Taizhou, Jiangsu, China; China Medical University, TAIWAN

## Abstract

Transportation is a major stressor in swine production, inducing endocrine imbalance, oxidative stress, and immune dysfunction. This study investigated the effects of Astragalus polysaccharides (APS) on transport-induced stress in piglets. Piglets were assigned to a control group (CON), a transport stress group (TS), and two APS-supplemented groups receiving low (APS-L) or high (APS-H) doses. Blood samples were collected on 1, 3, and 7 days post-transportation to assess stress hormones, oxidative status, and inflammatory responses. Compared with the CON group, piglets of TS group significantly elevated norepinephrine (NE) and epinephrine (EPI) levels, increased reactive oxygen species (ROS), reduced glutathione peroxidase (GSH-Px) activity, and upregulated pro-inflammatory cytokines (TNF-α and IL-6). APS supplementation attenuated these transport-induced changes. At 1 day after transportation, piglets in both APS-L and APS-H groups exhibited lower NE, EPI, ROS, TNF-α, and IL-6 levels and higher GSH-Px activity than those in the TS group. The APS-H group showed greater protective effects than the APS-L group. By 3 and 7 days, oxidative and inflammatory parameters in APS-treated piglets gradually normalized, with the APS-H group showing values more comparable to those of the CON group. These results indicate that APS alleviates transport-induced stress by modulating the endocrine response, reducing oxidative stress, and attenuating inflammatory responses. APS supplementation may serve as a nutritional strategy to improve the health and welfare of piglets during transportation.

## Introduction

Transportation is one of the most stressful procedures in modern swine production systems [1]. During transportation, piglets are exposed to multiple stressors such as crowding, fasting, vibration, and temperature fluctuations [2,3]. These stressors activate the sympathetic–adrenal–medullary (SAM) system, resulting in increased secretion of norepinephrine (NE) and epinephrine (EPI), which in turn induce oxidative stress and alter immune responses [4,5]. Such physiological changes negatively affect animal welfare, reduce growth performance, and compromise meat quality, ultimately leading to substantial economic losses [6].

Several management strategies have been investigated to alleviate transport stress in piglets, including improvements in vehicle design, reduction of transport duration, and nutritional interventions [7]. Among these approaches, natural feed additives with antioxidant and immunomodulatory properties have attracted increasing attention [8]. Astragalus polysaccharides (APS), extracted from *Astragalus membranaceus*, have been reported to possess multiple biological activities, including antioxidant, immunomodulatory, and anti-inflammatory effects [9,10]. Previous studies have demonstrated that APS possesses antioxidant and immunomodulatory activities across different animal species. For example, APS has been shown to improve antioxidant capacity and regulate immune function in weaned piglets [11]. Similarly, APS enhanced the efficacy of an inactivated Edwardsiella ictaluri vaccine by promoting immune responses in yellow catfish [12].

The effects of APS on transport-induced stress in piglets remain largely unknown. In particular, whether APS supplementation can modulate stress hormone secretion, relieve oxidative stress, and regulate inflammatory responses during transportation has not been fully investigated. Therefore, the aim of this study to investigate the protective effects of APS on piglets subjected to transport stress. The findings are expected to provide new insights into the application of APS as a natural feed additive for improving animal welfare and health under transport conditions.

## Materials and methods

### Animals and experimental design

A total of 32 healthy crossbred piglets (Duroc × Landrace × Yorkshire, average body weight 28 ± 2 kg) were obtained from the experimental farm of Jiangsu Academy of Agricultural Sciences. All piglets were confirmed to be clinically healthy and seronegative for major swine pathogens. This study was approved by the Ethics Committee of Animal Experiments center of Jiangsu Agri-Animal Husbandry Vocational College (Approval Number jsahvc-2025–8). All animal studies were approved by the Institutional Animal Care and Use Committee of Jiangsu Agri-Animal Husbandry Vocational College, and followed the National Institutes of Health guidelines for the performance of animal experiments. In addition, permission was obtained to conduct the study from the owner.

The piglets were randomly assigned into four groups (n = 8 per group), a control group (CON), a transport stress group (TS), a low-dose APS group (APS-L), and a high-dose APS group (APS-H). Piglets in the CON group remained in pens without transportation and were fed a basal diet. Piglets in the TS group were fed a basal diet and subjected to transportation for 6 h. Piglets in the APS-L (Low-dose APS) and APS-H (High-dose APS) groups were fed the basal diet supplemented with 200 mg/kg and 400 mg/kg APS, respectively, for 14 days before transportation, followed by 6 h of transportation. Blood samples were collected from piglets using standard procedureson, with every effort made to minimize handling time, stress, and discomfort. Because blood sampling was minimally invasive, no anesthesia or analgesia was administered. No animals were euthanized or sacrificed during the study.

APS (purity ≥ 90%, Solarbio, Beijing, China, Cat. No. SA9790) was used in this study. According to the manufacturer, the product is a water-soluble Astragalus polysaccharide extract with a purity of ≥90%. Detailed information regarding the monosaccharide composition and molecular weight distribution was not available from the manufacturer and has therefore been added as a limitation of the present study.

### Transportation procedure

Piglets in the TS, APS-L and APS-H groups were transported using a commercial livestock vehicle at a stocking density of 0.5 m^2^ per pig. The transportation lasted for 6 h on paved roads at an average temperature of 25–28 °C and a relative humidity of 60–70%. The vehicle was driven at a speed of 60–80 km/h. Feed and water were not provided during transportation.

### Sample collection

Blood samples (10 mL) were collected by anterior vena cava venipuncture. Piglets in the CON group were sampled once on day 0 without transportation. Piglets in the TS group were sampled once immediately after 6 h of transportation. For the APS-L and APS-H groups, blood samples were collected on 1, 3, and 7 days post-transportation. Serum was separated by centrifugation (3000 × g, 15 min, 4 °C) and stored at −80 °C until analysis.

### Determination of stress-related hormones and heat shock proteins

Serum concentrations of NE (ml002414), EPI (ml002318), heat shock protein 70 (HSP70, ml002377), and heat shock protein 90 (HSP90, ml025937) were determined using commercial ELISA kits (Shanghai Enzyme-linked Biotechnology Co., Ltd., Shanghai, China) according to the manufacturer's instructions.

### Oxidative stress and antioxidant indices

Serum samples were analyzed for reactive oxygen species (ROS, R&D Systems, Inc., NBP3–25793) and glutathione peroxidase (GSH-Px, Nanjing Jiancheng Bioengineering Research Institute, A005-1-2) using commercial assay kits.

### Cytokine determination

The concentrations of interleukin-6 (IL-6 ml025981) and tumor necrosis factor-α (TNF-α, ml002360) were determined using commercial ELISA kits (Shanghai Enzyme-linked Biotechnology Co., Ltd., Shanghai, China) according to the manufacturer's instructions.

### Statistical analysis

Data are presented as the mean ± standard deviation (SD). Statistical analyses were performed using SPSS 20.0 software. Differences among groups were analyzed by one-way analysis of variance (ANOVA) followed by the least significant difference (LSD) post hoc test for multiple comparisons. *P* value less than 0.05 was considered statistically significant.

## Results

### APS supplementation attenuated neuroendocrine responses to transport stress

**Fig 1:**
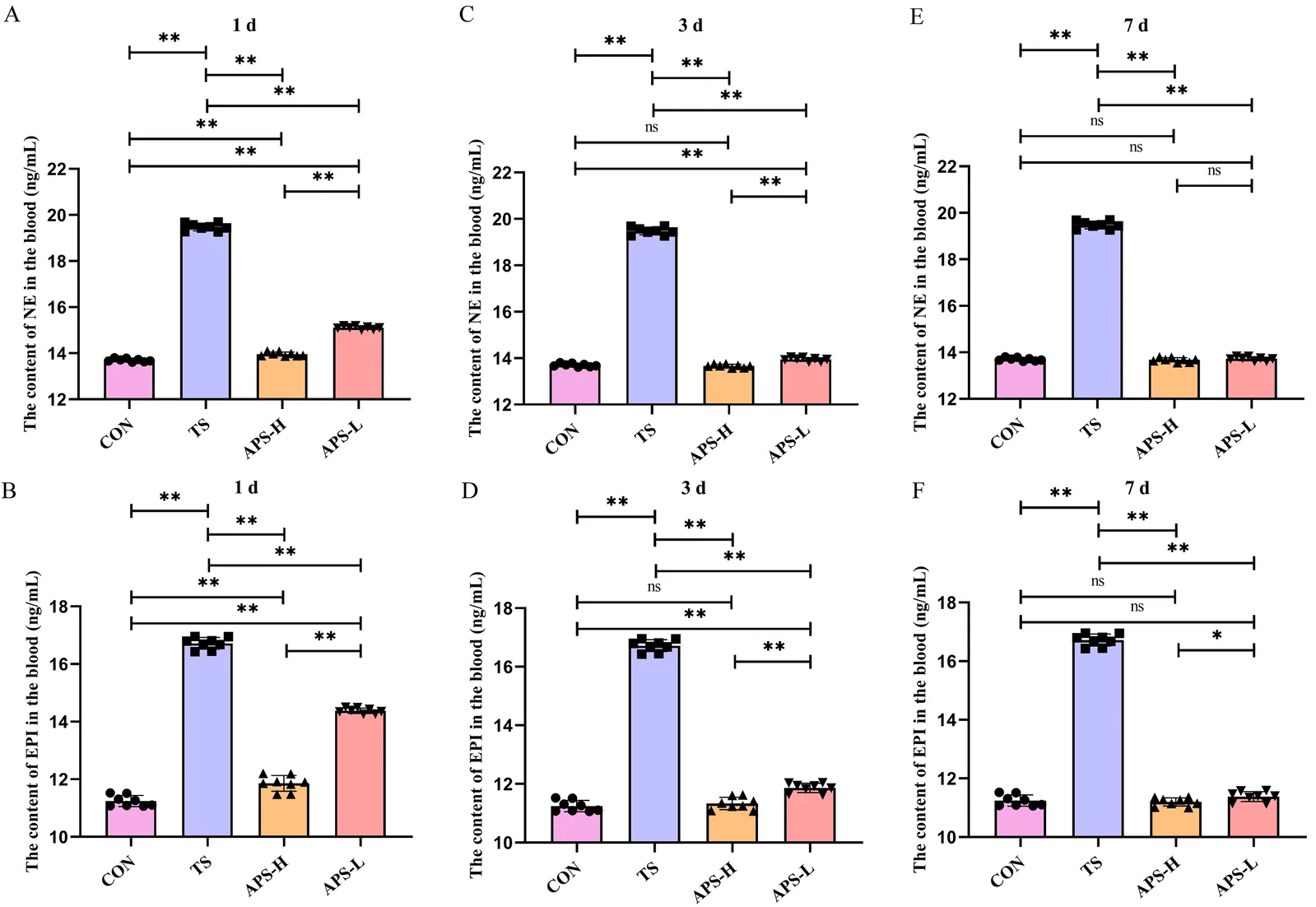
Effects of APS supplementation on serum NE and EPI concentrations in piglets following transportation stress. Data are presented as mean ± SEM (n = 8 per group). CON, non-transported control group; TS, transportation stress group; APS-L, transportation stress + 200 mg/kg APS group; APS-H, transportation stress + 400 mg/kg APS group. *P < 0.05 and **P < 0.01 indicate significant differences between groups.

Serum norepinephrine (NE) and epinephrine (EPI) concentrations were significantly higher in the TS group than in the CON group on 1, 3, and 7 days after transportation (Fig 1) (*P* < 0.01). APS supplementation significantly reduced serum NE and EPI concentrations compared with the TS group at all three time points (Fig 1) (*P* < 0.01). Furthermore, the APS-H group exhibited significantly lower NE and EPI concentrations than the APS-L group at 1 and 3 days after transportation (Fig 1A-1D) (*P* < 0.01). At 7 days after transportation, serum NE concentrations were not significantly different between the APS-L and APS-H groups, whereas serum EPI concentrations remained significantly lower in the APS-H group than in the APS-L group (Fig 1E-1F) (*P* < 0.05). In addition, serum NE and EPI concentrations in the APS-H and APS-L group were not significantly different from those in the CON group at 7 days after transportation (Fig 1E-1F). These results suggest that APS supplementation attenuated the transport-induced increase in serum NE and EPI concentrations, with the high-dose APS treatment producing a greater reduction than the low-dose treatment.

### APS reduced cellular stress markers (HSP70 and HSP90)

**Fig 2:**
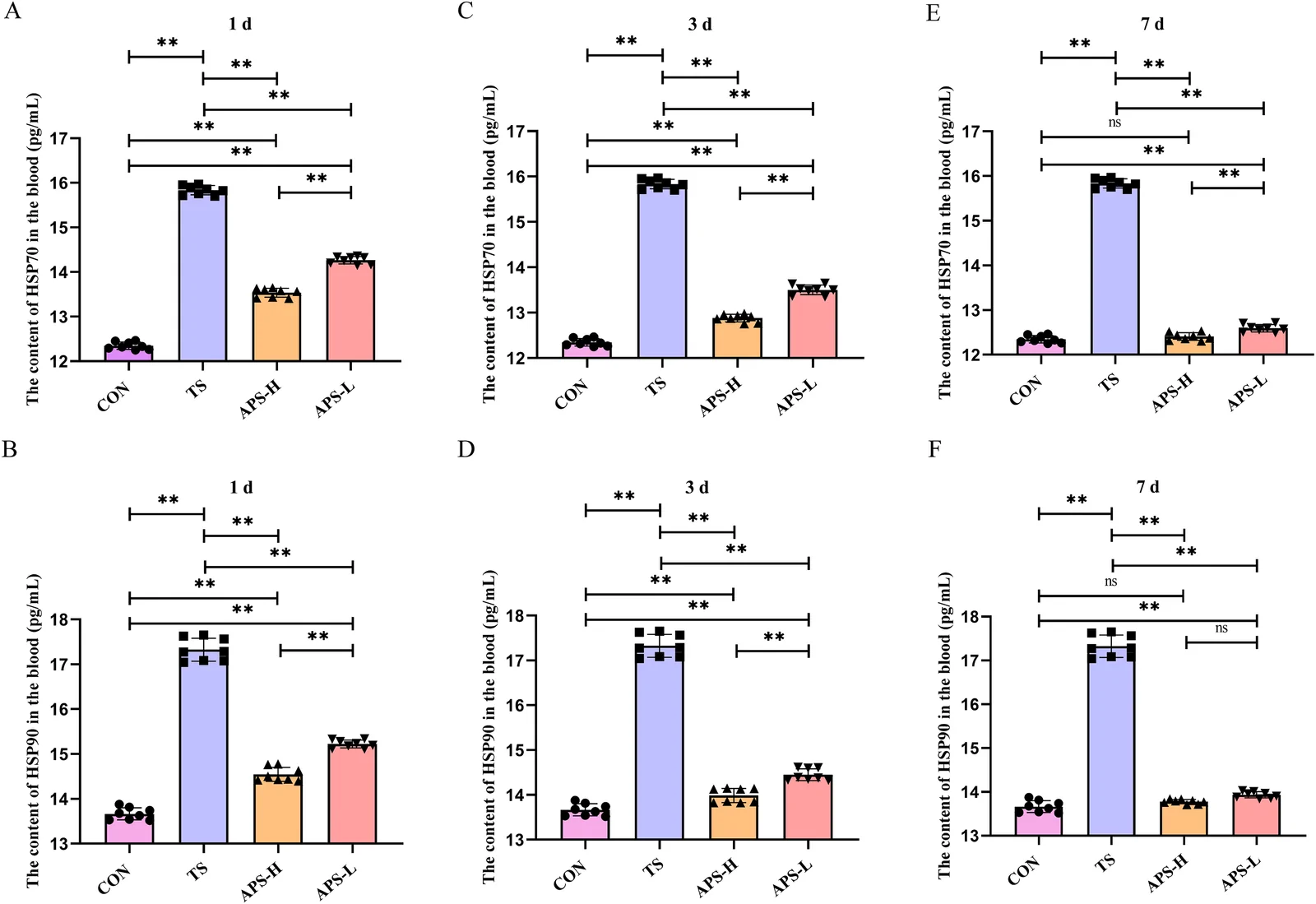
Effects of APS supplementation on serum HSP70 and HSP90 concentrations in piglets following transportation stress. Data are presented as mean ± SEM (n = 8 per group). CON, non-transported control group; TS, transportation stress group; APS-L, transportation stress + 200 mg/kg APS group; APS-H, transportation stress + 400 mg/kg APS group. **P < 0.01 indicate significant differences between groups.

As shown in Fig 2, serum HSP70 and HSP90 concentrations were significantly higher in the TS group than in the CON group on 1, 3, and 7 days after transportation (*P* < 0.01), indicating that transport stress induced a sustained heat shock response. APS supplementation significantly reduced serum HSP70 and HSP90 concentrations compared with the TS group at all three time points (*P* < 0.01). In addition, the APS-H group exhibited significantly lower HSP70 and HSP90 concentrations than the APS-L group at 1 and 3 days post-transportation (Fig 2A-2D) (*P* < 0.01). At 7 days after transportation, serum HSP70 and HSP90 concentrations in the APS-H group were not significantly different from those in the CON group, whereas the APS-L group still showed significantly higher concentrations than the CON group (Fig 2E-2F) (*P* < 0.01).

### APS improved antioxidant enzyme activities (ROS and GSH-Px)

**Fig 3:**
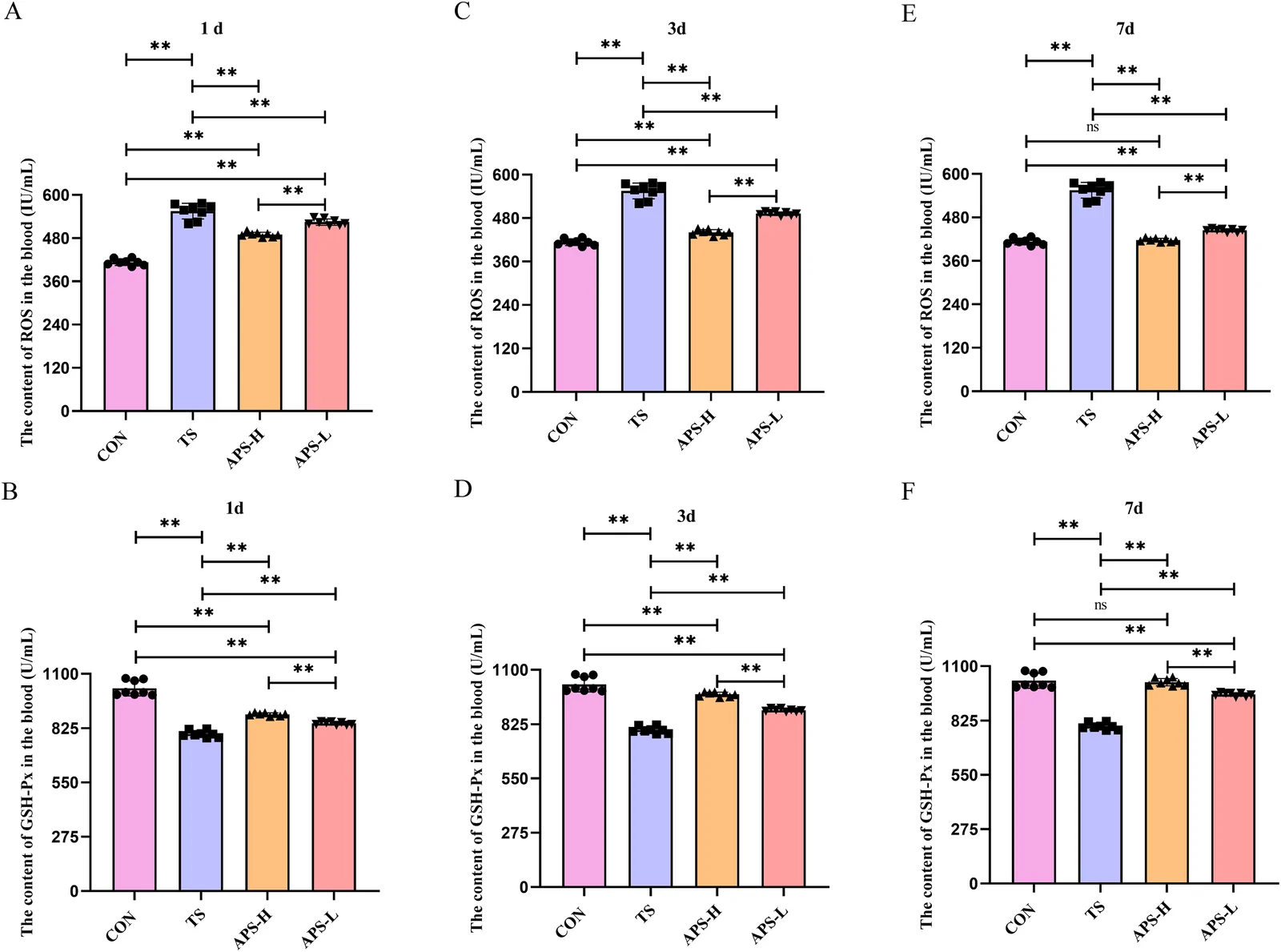
Effects of APS supplementation on serum ROS and GSH-Px levels in piglets following transportation stress. Data are presented as mean ± SEM (n = 8 per group). CON, non-transported control group; TS, transportation stress group; APS-L, transportation stress + 200 mg/kg APS group; APS-H, transportation stress + 400 mg/kg APS group. **P < 0.01 indicate significant differences between groups.

As shown in Fig 3, serum ROS levels were significantly higher, whereas GSH-Px activity was significantly lower, in the TS group than in the CON group on 1, 3, and 7 days after transportation (*P* < 0.01). APS supplementation significantly decreased serum ROS levels and increased GSH-Px activity compared with the TS group at all three time points (*P* < 0.01). Furthermore, the APS-H group exhibited significantly lower ROS levels and higher GSH-Px activity than the APS-L group (Fig 3A-3F) (*P* < 0.01). At 7 days after transportation, serum ROS levels and GSH-Px activity in the APS-H group were not significantly different from those in the CON group, whereas the APS-L group still differed significantly from the CON group (Fig 3E-3F) (*P* < 0.01). These results indicate that APS supplementation alleviated transport-induced oxidative stress, with the high-dose APS treatment producing greater improvements than the low-dose treatment.

### APS attenuated pro-inflammatory cytokine responses (TNF-α and IL-6)

**Fig 4:**
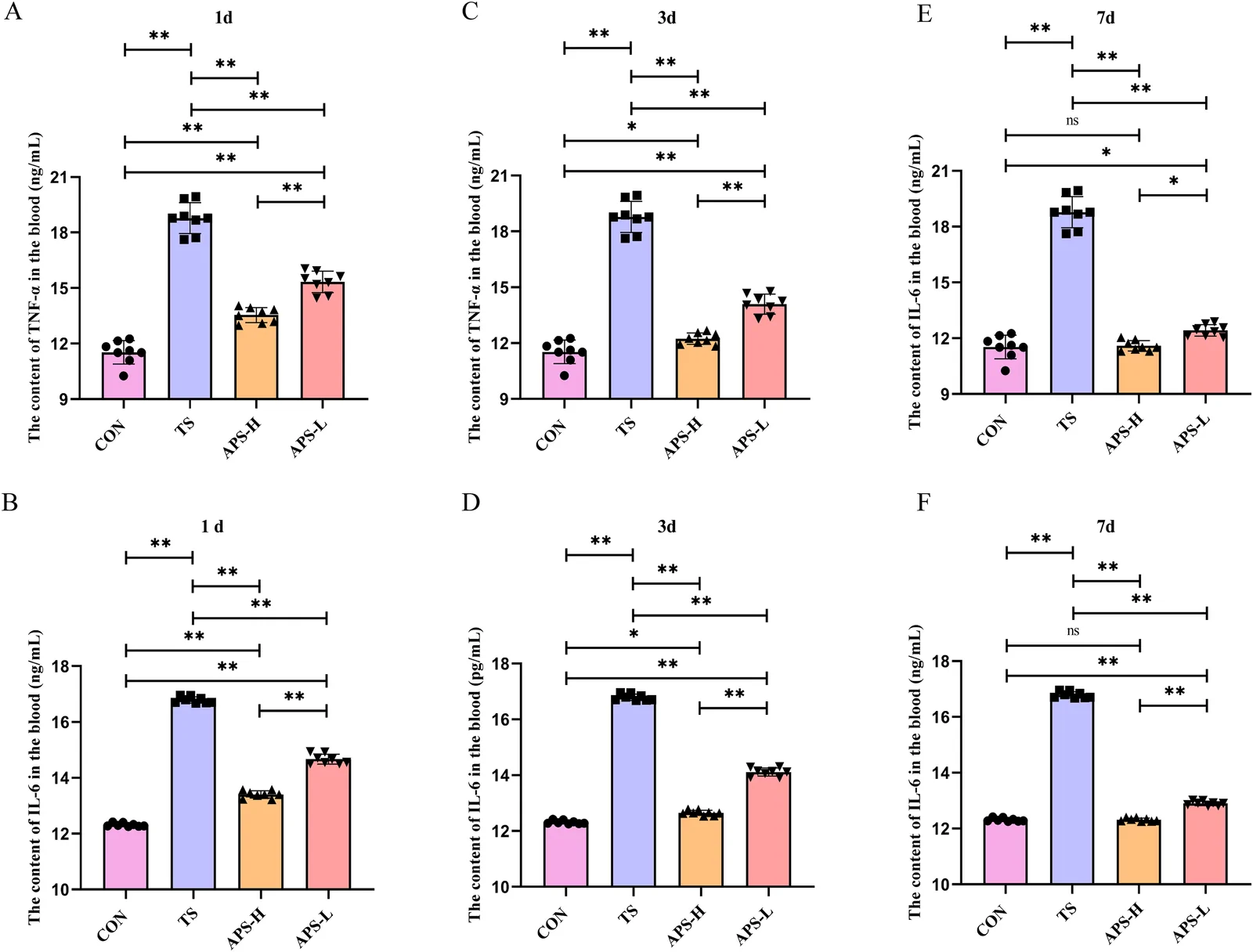
Effects of APS supplementation on serum TNF-α and IL-6 concentrations in piglets following transportation stress. Data are presented as mean ± SEM (n = 8 per group). CON, non-transported control group; TS, transportation stress group; APS-L, transportation stress + 200 mg/kg APS group; APS-H, transportation stress + 400 mg/kg APS group. *P < 0.05 and **P < 0.01 indicate significant differences between groups.

Serum tumor TNF-α and IL-6 concentrations were significantly higher in the TS group than in the CON group on 1, 3, and 7 days after transportation (Fig 4A-4F) (*P* < 0.01). APS supplementation significantly reduced serum TNF-α and IL-6 concentrations compared with the TS group at all three time points (Fig 4A-4F) (*P* < 0.01). Furthermore, the APS-H group exhibited significantly lower TNF-α and IL-6 concentrations than the APS-L group at most time points (Fig 4A-4F) (*P* < 0.05 or *P* < 0.01). At 7 days after transportation, serum TNF-α and IL-6 concentrations in the APS-H group were not significantly different from those in the CON group (Fig 4E-4F). In contrast, both TNF-α and IL-6 concentrations in the APS-L group remained significantly higher than those in the CON group (Fig 4E-4F) (*P* < 0.05 or *P* < 0.01). These results indicate that APS supplementation attenuated the transport-induced inflammatory response, with the high-dose APS treatment producing greater reductions in pro-inflammatory cytokine concentrations than the low-dose treatment.

## Discussion

Transportation is widely recognized as a major stressor in swine production, leading to profound neuroendocrine activation, cellular stress, oxidative imbalance, and inflammatory responses [6]. In the present study, piglets subjected to transportation exhibited significantly elevated serum NE, EPI, HSP70, HSP90 TNF-α and IL-6 concentrations, together with increased ROS levels and decreased GSH-Px activity. These results confirm the acute stress response induced by transportation, consistent with previous studies in piglets and cattle [13,14].

Importantly, Astragalus polysaccharides (APS) supplementation alleviated the transport-induced changes in serum NE and EPI concentrations. Both APS-L and APS-H reduced NE and EPI levels at 1 day post-transportation, suggesting that APS mitigates activation of the SAM system [15,16]. However, the APS-H group exhibited faster normalization of catecholamine levels by 3 days, whereas APS-L remained elevated until 7 days, indicating stronger neuroendocrine modulation at higher APS dosage [17].

APS supplementation also reduced serum HSP70 and HSP90 concentrations following transportation. HSP70 and HSP90 are stress-inducible molecular chaperones that play important roles in maintaining protein homeostasis under stressful conditions, and their increased expression is widely regarded as a marker of cellular stress [18,19]. APS-H supplementation suppressed HSP induction more effectively than APS-L, and values in APS-H piglets returned to baseline by 7 days, demonstrating enhanced cellular resilience at the higher dose [20,21].

Oxidative stress is a common feature of transport stress, evidenced by increased ROS levels and decreased GSH-Px activity [22]. APS supplementation preserved antioxidant defense capacity. Previous studies have reported that APS enhances antioxidant capacity, potentially through regulation of the nuclear factor erythroid 2-related factor 2 (Nrf2) signaling pathway [23,24]. In the present study, Compared with the APS-L group, the APS-H group showed greater improvement in oxidative stress status. These results highlight the importance of an adequate APS dosage in maintaining redox balance. In addition, APS supplementation also reduced the release of pro-inflammatory cytokines (TNF-α and IL-6), which are known to be upregulated during transport stress and contribute to systemic inflammation and tissue injury [25,26]. Compared with the APS-L group, the APS-H group showed greater attenuation of the transport-induced inflammatory response, as reflected by lower TNF-α and IL-6 concentrations. This indicates that APS not only mitigates acute inflammation but also accelerates the resolution phase, particularly at higher doses.

Collectively, our findings demonstrate that APS supplementation confers multi-faceted protection against transport stress in piglets through modulation of neuroendocrine activity, suppression of cellular stress, enhancement of antioxidant defenses, and inhibition of inflammatory cytokines. APS supplementation exerted protective effects against transport-induced stress, with the high-dose APS (400 mg/kg) showing superior efficacy compared with the low dose (200 mg/kg). These observations are consistent with reports that APS exerts immunomodulatory and antioxidative activities in livestock. From a practical perspective, APS supplementation at higher doses may represent an effective nutritional strategy to enhance stress resilience in piglets during transportation. This could be beneficial for improving physiological stress adaptation under commercial production conditions. Future studies should explore the underlying molecular mechanisms, including NF-κB, MAPK, and Nrf2 signaling pathways, to further elucidate the protective mechanisms of APS against transport-induced stress. A limitation of the present study is that only short-term physiological and protein-level responses were assessed, while long-term production performance parameters, such as growth rate and meat quality, were not included. Therefore, further research is needed to evaluate the effects of different APS dosages on both mechanistic pathways and long-term production outcomes under commercial conditions.
